# The Toxicity and Benefit of Various Dosing Strategies for Interleukin-2 in Metastatic Melanoma and Renal Cell Carcinoma

**Published:** 2015-05-01

**Authors:** Laura A. Pachella, Lydia T. Madsen, Joyce E. Dains

**Affiliations:** University of Texas MD Anderson Cancer Center, Houston, Texas

## Abstract

Interleukin-2 (IL-2) therapy has been used with success in curing meta­static renal cell carcinoma and melanoma in a small minority of patients. However, the benefits can be accompanied by severe toxicity. This review of the literature discusses varying doses of IL-2 and their associated re­sponse rates and the toxicities associated with treatment. The review also explores the maximally beneficial dose with the most tolerable side effects. Although the higher-dose regimens with a more frequent dosing schedule produce higher-grade toxicity, they were found to deliver the most durable and complete responses. It is recommended to use a higher-dose regimen (720,000 IU/kg every 8 hours for a maximum of 15 doses) and provide sup­portive care for toxicity, so patients can have maximal benefit from therapy.

The administration of high-dose intravenous interleukin-2 (IL-2) for metastatic renal cell carcinoma and metastatic melanoma was first approved by the US Food and Drug Administration (FDA) in 1992 and 1998, respectively (Spanknebel et al., 2005). Interleukin-2 is a cytokine that stimulates the body’s immune system to recognize, target, and destroy cancer cells; it differs from conventional chemotherapy, which works by killing cancer cells directly. Interleukin-2 was discovered in 1975 as a growth-promoting cytokine for bone marrow–deprived T lymphocytes, its most prominent function (Gaffen & Liu, 2004). Although it remains unclear how IL-2 induces an anticancer response in the body, it is hypothesized that the exogenous IL-2 may promote a cytotoxic T-lymphocyte–mediated antitumor response (Gaffen & Liu, 2004).

The use of high-dose IL-2 in renal cell carcinoma and melanoma are two rare instances in oncology where an effective treatment has been identified to potentially cure a widely metastatic solid tumor (Gaffen & Liu, 2004). The use of IL-2 has been shown to lead to a complete tumor response and durable long-term survival in a small percentage of patients (Klapper et al., 2008). In renal cell carcinoma, a tumor regression rate of 20% and a complete response rate of 9% have been reported (Gaffen & Liu, 2004). In melanoma, the tumor regression rate was slightly lower at 17%, with a complete response rate of 7% (Gaffen & Liu, 2004).

There are three identified classes of IL-2 receptor complexes, with a high, intermediate, or low affinity for binding with IL-2 (Gaffen & Liu, 2004). It can be extrapolated that different routes and doses of IL-2 may selectively enhance the effects on high- or low-affinity IL-2 receptors. A high serum level of IL-2 may saturate receptors and allow for a greater T-lymphocyte response against the tumor; therefore, high-dose regimens were created empirically as anticancer treatment. The effects of high- and low-dose IL-2 may be mediated by the affinity level of receptors; however, dosing schemas were created prior to the discovery and understanding of the receptor subunits (Gaffen & Liu, 2004).

Varying levels of systemic toxicity are expected in patients who receive IL-2. There is evidence that increased doses of IL-2 lead to increased toxicity, which is not tolerable to all patients (Schwartzentruber, 2001). Side effects can limit the duration of treatment as well as interfere with patient safety if not managed by skilled clinicians (Schwartzentruber, 2001).

The current FDA-approved dose of high dose IL-2 is 600,000 IU/kg per dose administered intravenously every 8 hours for a maximum of 14 doses on days 1 to 5 (cycle 1) and days 15 to 19 (cycle 2), with a maximum of 28 doses for 1 course (FDA, 2011, p. 15). The dosing is discontinued when the patient has reached a dose-limiting toxicity that would compromise the patient’s safety.

Several dosing schemes, which include intravenous high dose (720,000 or 600,000 IU/kg), low-dose subcutaneous injections, and IL-2 in combination with other treatments, have been used in practice for maximal therapeutic benefit. The variety of regimens makes it difficult to determine the most effective and least toxic option for the treatment of metastatic renal cell carcinoma and melanoma. There are currently 60 institutions in North America that administer high-dose IL-2 therapy for metastatic melanoma and renal cell carcinoma (Dutcher et al., 2014).

Therefore, the purpose of this comprehensive review is to examine the use of IL-2 at different dosing regimens in metastatic renal cell carcinoma and melanoma to determine whether there is a target dose that produces optimal patient outcome with minimal toxicity.

## METHODS

We identified relevant articles in the Scopus and PubMed databases utilizing the search terms "interleukin-2," "IL-2," "toxicity," "response," "melanoma," and "renal cell carcinoma." There were 363 results that were populated. Limitations imposed within the search were for articles published between January 2002 and October 2014, English language, peer-reviewed journal articles, and human populations. Articles were excluded if upon review they were not clinically relevant to adult patients receiving IL-2 for renal cell carcinoma or melanoma. Case studies and review articles were excluded, as were articles that focused specifically on one area of metastasis. Studies that focused on one population (geriatrics or pediatrics) were also excluded. This comprehensive review includes 10 articles that met these search criteria. Table 1 details the studies included.

**Table 1:**
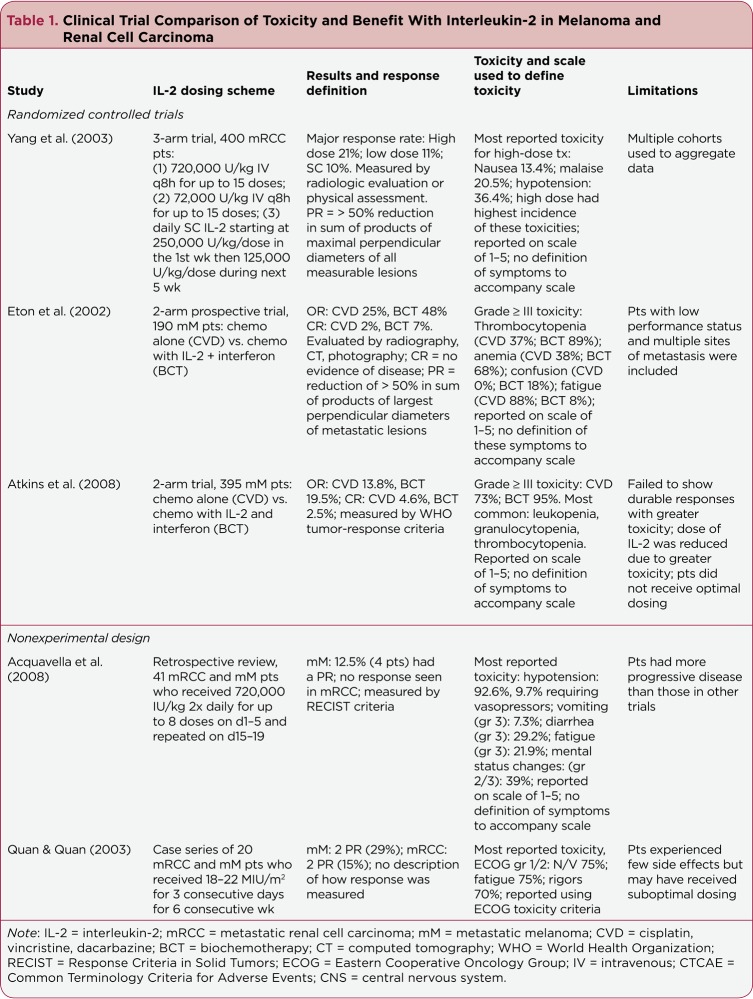
Clinical Trial Comparison of Toxicity and Benefit With Interleukin-2 in Melanoma and Renal Cell Carcinoma

**Table 1b:**
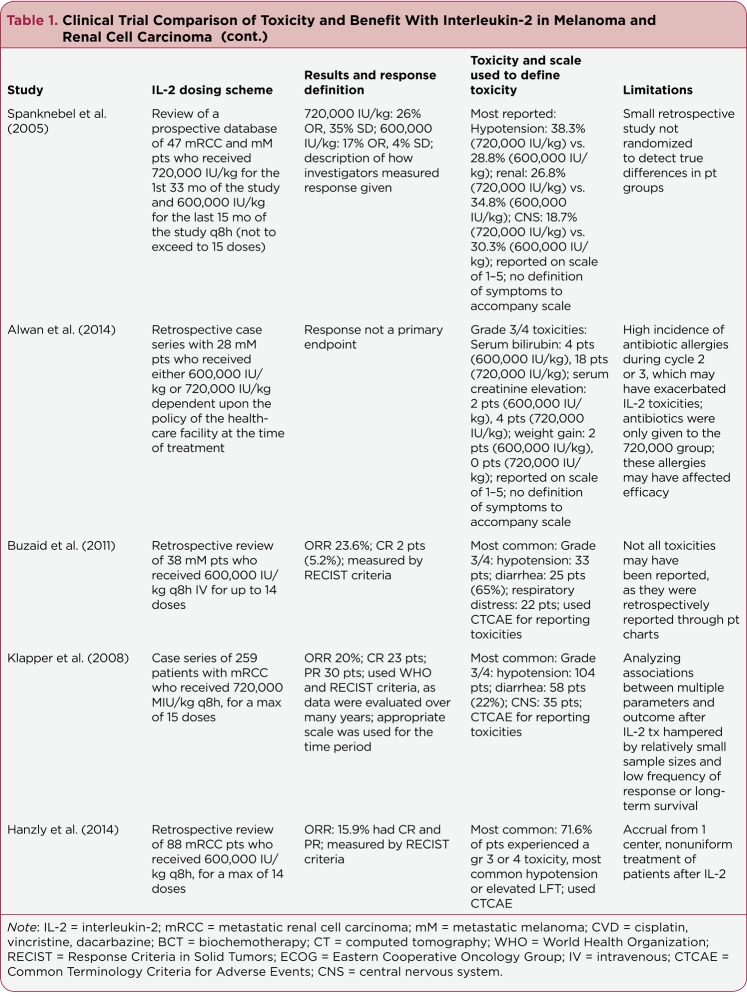
Clinical Trial Comparison of Toxicity and Benefit With Interleukin-2 in Melanoma and Renal Cell Carcinoma (cont.)

The data extracted from the articles include the protocol for administering IL-2 with the dosing schema. The reported toxicities and response rates were also extrapolated. The scales and measures for reporting toxicities and response were evaluated for their validity.

## RESULTS

**Patient Toxicities**

Common toxicities of high-dose intravenous IL-2 are included in Table 2. Lymphoid infiltration has been observed in the histology of many organs and may contribute to some of these manifestations (Schwartzentruber, 2001). Prophylaxis and supportive care during administration and in the immediate period after treatment are essential. Renal failure is of greatest concern in renal cell patients, as they may be post nephrectomy and at greater risk with only one remaining kidney (Hanzly et al., 2014).

**Table 2:**
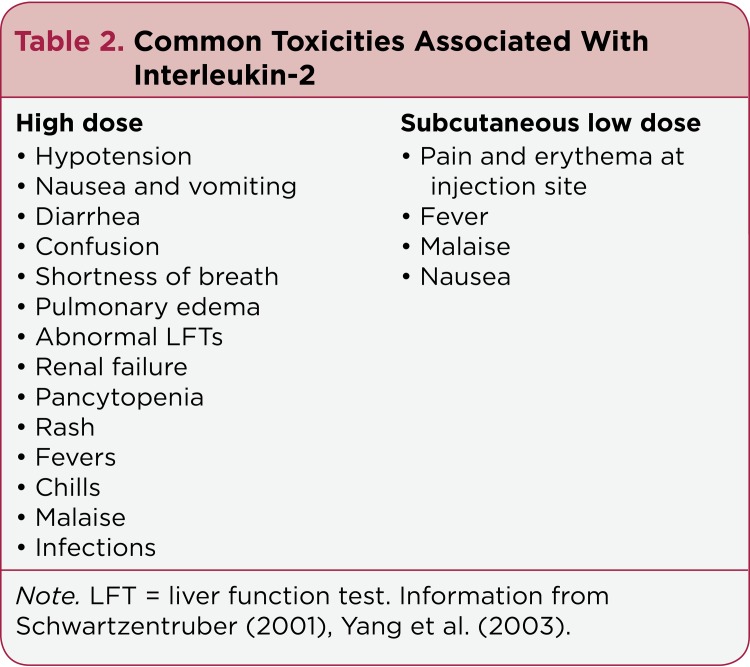
Common Toxicities Associated With Interleukin-2

Capillary leak syndrome is a life-threatening toxicity resembling septic shock that may occur with intravenous high-dose IL-2. The increased IL-2 in the circulation and immune stimulation cause may massive cytokine release and inflammatory reaction. Capillaries become more permeable, leading to the loss of intravascular fluid into extravascular spaces (Mavroukakis, Muehlbauer, White, & Schwartzentruber, 2001). Possible clinical presentations of capillary leak syndrome are listed in Table 3. Interleukin-2 may also disturb and impair neutrophil function, which may increase the risk of bacterial infection (Alwan et al., 2014). Given the severe side-effect profile with high-dose IL-2, prudence and expert clincial judgment are necessary when selecting patients for treatment.

**Table 3:**
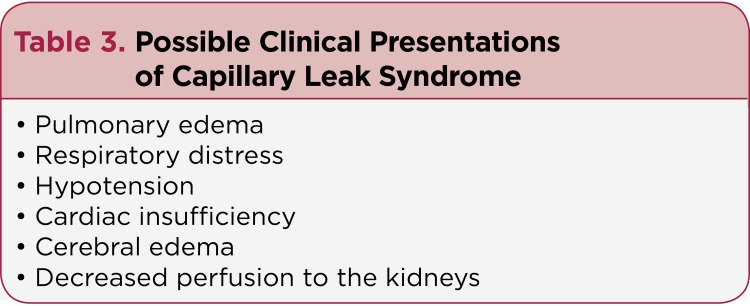
Possible Clinical Presentations of Capillary Leak Syndrome

There was no standardized tool consistently used to assess toxicity in the studies under examination. Klapper et al. (2008), Buzaid et al. (2011), and Hanzly et al. (2014) applied the Common Terminology Criteria for Adverse Events (CTCAE) for grading and reporting toxicity. The standardized CTCAE scale clearly defines side effects from any treatment and the grade corresponding to those symptoms. Quan and Quan (2003) used the Eastern Cooperative Oncology Group (ECOG) toxicity criteria in reporting adverse events. Yang et al. (2003), Spanknebel et al. (2005), Alwan et al. (2014), Acquavella et al. (2008), Eton et al. (2002), and Atkins et al. (2008) all reported toxicity on a graded scale from 1 to 5 without reference to a standardized scale.

Hypotension, as a result of capillary leak syndrome, was one of the most commonly reported side effects from IL-2 treatment among the studies. Acquavella et al. (2008) reported a rate of hypotension as high as 92.6% in patients receiving 720,000 IU/kg, with 9.7% requiring vasopressor support. Spanknebel et al. (2005) reported an increase in hypotension by 10% when comparing 720,000 IU/kg with 600,000 IU/kg. Klapper et al. (2008) noted that hypotension was the most common grade 3 or 4 toxicity among patients receiving 720,000 IU/kg.

Gastrointestinal symptoms, including diarrhea, nausea, and vomiting, were reported (Mavroukakis et al., 2001). Buzaid et al. (2011) investigated a dosing regimen of 600,000 IU/kg three times a day and reported that 65% of patients had grade 3 or 4 diarrhea. When Acquavella et al. (2008) used the dosing regimen of 720,000 IU/kg twice a day, 29.2% of patients reported grade 3 diarrhea. Klapper et al. (2008) examined 720,000 IU/kg three times daily, with grade 3 or 4 diarrhea reported in 22% of patients.

Neurologic side effects of IL-2 have been previously reported to present as lethargy, anxiety, vivid dreams, confusion, sleep disturbance, decreased concentration, mood swings, hallucinations, depression, and coma (Mavroukakis et al., 2001). Spanknebel et al. (2005) reported a lower rate of central nervous system (CNS) toxicity in patients receiving 720,000 IU/kg (18.7%) as opposed to 600,000 IU/kg (30.3%). Acquavella et al. (2008) looked at the 720,000 IU/kg dosing regimen and reported 39% of patients had grade 2 or 3 CNS toxicity. Central nervous system toxicity was correlated by Yang et al. (2003) based on the amount of dose received, with 10.2% of high-dose patients (720,000 IU/kg), 3.7% of low-dose patients (72,000 IU/kg), and 1.7% of those receiving subcutaneous dosing reporting grade 3 or 4 neurotoxicity.

The side effects associated with low-dose (72,000 IU/kg) intravenous treatment were not trivial; more than half of the treatment courses were discontinued due to intolerable side effects. The two most common reasons for discontinuation of low-dose intravenous treatment were hypotension and patient refusal (Yang et al., 2003).

Adding IL-2 to chemotherapy (biochemotherapy, BCT) has proved to be more toxic than chemotherapy in two trials. Atkins et al. (2008) showed grade 3 or greater toxicity on the BCT arm in 95% of patients. By the fourth cycle of therapy, only 57% of patients were able to receive a full dose of IL-2 due to side effects, thus compromising the potential effectiveness of the drug regimen. Toxicities included leukopenias, hypotension, and metabolic dysfunction (Atkins et al., 2008). Eton et al. (2002) reported that patients who received BCT were twice as likely to have grade 3 or 4 thrombocytopenia or anemia as the chemotherapy group. Both studies dosed IL-2 at 9 million IU/m2 as continuous infusions for 4 days.

Three studies reported patient death attributable to IL-2 therapy. Atkins et al. (2008) reported deaths among patients receiving BCT, related to infection and renal failure. Eton et al. (2002) reported mortality in one BCT patient, subsequent to CNS hemorrhage and renal failure. It should be noted, however, that two of the deaths reported by Klapper et al. (2008) occurred over 25 years ago, at the beginning of the clinical use of 720,000 IU/kg of IL-2 when risk and the need for supportive care measures were not clearly delineated.

**Patient Outcomes**

The primary outcome of response to treatment was defined by various endpoints such as disease-free progression, total survival, or factors correlated with response. In solid tumors, such as melanoma and renal cell carcinoma, a standard to measure response to treatment is computed tomography (CT) or magnetic resonance imaging (MRI). Tumor measurement by radiologists requires a standardized method and documentation to allow for reproducibility.

Criteria for measuring response varied from study to study. The World Health Organization (WHO) tumor response criteria were widely used to standardize independent image review for oncology studies. Subsequently, Response Criteria in Solid Tumors (RECIST) was considered to better refine measurement criteria that could be compared between studies (Therasse et al., 2000). The RECIST criteria are currently used most frequently for evaluating response in solid tumors. The WHO tumor response criteria were used in the study by Atkins et al. (2008). The RECIST criteria were used by Acquavella et al. (2008), Buzaid et al. (2011), and Hanzly et al. (2014). And Klapper et al. (2008) used both the WHO and RECIST criteria, as the data spanned many years; the respective scale was used for each time period.

In the data reported by Spanknebel et al. (2005), Yang et al. (2003), and Eton et al. (2002), the criteria for grading response were reported but without a standardized scale, creating difficulty with reproducing the findings in subsequent studies. In the article by Quan and Quan (2003), the results were reported through the use of scales; however, there was no description given of how these findings were calculated.

High-dose IL-2 is a regimen ranging from 600,000 IU/kg to 720,000 IU/kg every 8 hours, and a low-dose IL-2 regimen is anything below this dosing protocol. Low-dose therapy was shown to have some response in patients; however, it rarely led to a complete response. Yang et al. (2003) described 2 of 93 patients who had complete and durable responses on daily subcutaneous injections 5 days a week beginning at a dose of 250,000 IU/kg in the first week and then 125,000 IU/kg during the next 5 weeks. Yang et al. (2003) also reported 7 patients of 241 who completely responded to intravenous IL-2 at a dose of 72,000 IU/kg.

Quan and Quan (2003) reported partial or minor responses in 6 of 20 patients who received low-dose intravenous IL-2 (18–22 MIU/m₂) as outpatients, with one dose daily for 3 days a week. Acquavella et al. (2008) reported 4 partial responses in the 41 patients who received high dose IL-2 (720,000 IU/kg); this group of patients received a more conservative schedule of twice-daily IL-2, for a total goal of eight doses. Both regimens did not produce any durable and sustained response, supporting Yang et al.’s conclusion that the administration regimen of high-dose IL-2 every 8 hours produces higher rates of response (Yang et al., 2003).

When chemotherapy alone was compared with chemotherapy and IL-2, no survival advantage for the BCT arm was observed (Atkins et al., 2008). However, Eton et al.’s comparison of chemotherapy and BCT demonstrated a major response rate, defined as a partial response in addition to a complete response, of 48% for BCT and 25% for chemotherapy alone. The complete response rate was 7% for BCT and 2% for chemotherapy (Eton et al., 2002).

In the comparison of two high-dose therapy regimens (720,000 IU/kg and 600,000 IU/kg), Spanknebel et al. (2005) reported 26% of patients in the higher-dosing group had objective responses on imaging compared with 17% in the lower-dose group. The initial overall response rate of 23% for all patients showed no statistically significant difference between dosing regimens. However, patients who received the higher dose of treatment were more likely to have stable disease after one course of IL-2. A criterion for continuing IL-2 treatment was stable disease, making patients who were given the higher dose more likely to receive subsequent cycles of IL-2 (Spanknebel et al., 2005).

In a review of 259 patients, Klapper et al. (2008) reported a total of 23 patients with a complete response at 720,000 IU/kg dosing and 30 patients with a partial response. The overall response rate was 20%. Some patients had an ongoing and durable response up to 18 years after initial treatment. On regression analysis, the only correlation to response was the cumulative dose given during the initial course (Klapper et al., 2008).

Buzaid et al. (2011) reported an overall response rate of 23.6% achieved in patients who received 600,000 IU/kg IL-2 after disease progression on BCT. The authors suggested there may be a benefit of IL-2 in this subgroup of patients (Buzaid et al., 2011). Of 39 patients, 2 achieved a complete and durable response.

Yang et al. (2003) reported a major response rate at 21%, a complete response rate of 6% with high dose IL-2 (720,000 IU/kg). Overall survival increased in this study when high- dose and low-dose outcomes were compared. Patients who tolerated doses of IL-2 without significant toxicity limiting its administration were able to receive more cumulative doses, which may be associated with a higher frequency of response (Yang et al., 2003).

Hanzly et al. (2014) described a complete response rate of 4.5% (4 of 88 patients) in a prospective study of metastatic renal cell patients who received IL-2. Additionally, 11.4% (10 of 88 patients) achieved a partial response. Hanzly and colleagues (2014) correlated response with IL-2 being the first-line therapy as opposed to patients first being exposed to other agents. Patients who were treated first with other therapies were noted to have a lower overall response rate as opposed to those treated with first-line IL-2. The majority of patients (92%) in this study had clear cell pathology, who were more likely to have a response.

## DISCUSSION

In comparing dosages of IL-2, the goal is to determine a dose that will provide the most benefit with the least amount of toxicity. For a small group of patients for whom cure is possible from metastatic disease, IL-2 is promising; however, in attempting to reduce toxicity, some regimens may negate the possibility of response. Our review of the literature suggests that high-dose IL-2 with supportive care for acute toxicity in the period immediately after its administration is the optimal dosing regimen with the most durable and complete response rates.

However, it is difficult to evaluate a durable complete cure rate when rates are below 10% and lesser benefit is not evidenced by a significant proportion of the remaining patients (Yang et al., 2003). Much of the data in these studies were retrospective and involved chart review to determine the side-effect profile; therefore, some of the information may have been omitted (Buzaid et al., 2011). The lack of standardization in scales used to measure adverse events does not allow for complete comparison between studies.

Virtually all toxicity seen with intravenous administration of high-dose IL-2 is reversible and dose-limited (Yang et al., 2003). Changes in dosing failed to improve quality-of-life assessments in patients on varying regimens of IL-2. The daily subcutaneous injections produced minor but chronic side effects, seen as more of an inconvenience than the short-lived severe toxicities of high-dose intravenous therapy (Yang et al., 2003).

There is no evidence to support a correlation between the severity of side effects and a higher propensity to respond to treatment (Gaffen & Liu, 2004). Alwan et al. (2014) did not look at response as a primary outcome; however, their findings recommend the standard use of 600,000 IU/kg, as a decrease in overall toxicity was seen. This is in contrast to the work by Yang et al. (2003), which did measure toxicity and survival outcome and found that the high-dose regimen (720,000 IU/kg) was associated with a rate of response when compared directly with the lower-dose regimens (72,000 IU/kg; Yang et al., 2003). Spanknebel et al. (2005) reported that patients who experienced a major objective response after a single course of high-dose IL-2 (720,000 IU/kg) seemed to have ongoing prevention of progressive disease.

Atkins et al. (2008) reported that BCT compared with chemotherapy alone caused a higher rate of grade 3 and 4 toxicities. The majority of these incidents were expected and reversible with the cessation of each cycle when detected and treated by skilled providers (Eton et al., 2002).

Atkins et al. (2008) concluded that BCT cannot be considered the standard of care for advanced melanoma because it failed to produce any durable responses. Eton et al. (2002) found major response rates of 48% with BCT and 25% with chemotherapy, while Atkins et al. reported a complete response rate of 2.5% for BCT and 4.6% for chemotherapy.

The differences between studies may be due to differences in methodology, including the fact that Atkins et al. (2008) discontinued or lowered the dose of IL-2 due to toxicities. The eligibility criteria for Eton et al.’s (2002) study were less selective, allowing for patients with a poor performance status and multiple organ involvement, including brain metastasis (Eton et al., 2002). On the basis of response rates, Eton et al. (2002) recommended that research and clinical treatment with BCT be continued.

There may be practical reasons why high-dose IL-2 is not favored at particular institutions. The administration of high-dose IL-2 requires a monitored setting and possible transfer to an intensive care unit, a potential disadvantage to hospital management, as it requires more resources than routine cancer treatment (Acquavella et al., 2008). The acuity of patient care is a serious concern, as extra hospital staff may be required to meet the demands of a treatment with highly complex adverse effects.

Although very low-dose regimens may circumvent some toxicity, there is appropriate concern that reducing the schedule may limit response rates or response durations (Quan & Quan, 2003). It is important to consider the balance between toxicity and dose intensity. A low dose can be given to patients with a poor performance status; however, this has not translated into clinical response. It is possible that low-dose IL-2 does not affect the high-affinity receptors, and this could explain why some patients had partial responses. It is unknown whether they could have had a complete response if they had received a higher dose regimen.

Additionally, there is great variation in the toxicities that present in IL-2 patients, as some experience very few side effects (Schwartzentruber, 2001). There may be some benefit for the low-dose regimens, as Yang et al. (2003) reported complete responses in a minority of patients who received subcutaneous or intravenous low-dose IL-2. However, no algorithm currently exists to identify patients who could be cured with low-dose therapy.

## CONSIDERATIONS FOR ADVANCED PRACTITIONERS

Advanced practitioners may screen patients for appropriate therapy in the outpatient setting and assess patients at the bedside for administration of IL-2. The recognition of expected adverse reactions and proper interventions to prevent extreme toxicities are vital pieces of therapy (Mavroukakis et al., 2001). The administration of IL-2 is considered safe when clinically appropriate patients are cared for by trained providers and nurses (Schwartzentruber, 2001). Advanced practitioners should recognize that treatment must be individualized; the clinical course may change rapidly, depending on how the patient responds to IL-2 (Schwartzentruber, 2001). Advanced practitioners should play a role in discharge planning, assuring the patient and family that the adverse events of IL-2 are dose-limited and should resolve after discharge from the hospital (Dutcher et al., 2014).

## CLINICAL IMPLICATIONS

To determine the optimal dosing of IL-2, a randomized controlled trial is necessary to overcome bias and detect a difference in responders and nonresponders, as the percentage of patients who receive benefit from this treatment remains low. Alwan et al. (2014) recommend multi-institutional accrual of patients with central radiology review.

Additionally, little is known about prognostic factors that can be linked to IL-2 response. Further research is needed to understand how IL-2 produces a complete and durable response in a small group of patients with metastatic melanoma and renal cell carcinoma and fails to stop even disease progression in others. There are currently no biomarkers or prognostic factors that have been identified to determine patients who may receive maximal benefit from high-dose IL-2 therapy, an important consideration when subjecting a patient to a treatment with profound side effects (Alwan et al., 2014). There is great interest in the results of the ongoing SELECT trial, which is evaluating the tumor-associated predictors of responsiveness to IL-2 (Hanzly et al., 2014).

The treatment for melanoma has included several new immunotherapies, each bringing with it a host of adverse effects. Adverse events related to these agents were reviewed by Ma and Armstrong (2014), and unique adverse effects related to ipilimumab (Yervoy), vemurafenib (Zelboraf), interferon, dacarbazine, and IL-2 were found. This is noteworthy for advanced providers to be aware of the adverse effects that may be associated with therapies used as alternatives to IL-2; patients may still find side effects or toxicities intolerable, limiting their ability to receive full dosing (Ma & Armstrong, 2014).

In recent years, targeted therapy has become more prevalent in oncology, and agents have been developed to treat melanoma and renal cell carcinoma. As a result, discussions on the value of IL-2 in comparison with these more focused treatments have emerged. Tyrosine kinase inhibitors used in renal cell carcinoma have increased the median survival by 10 months but have failed to produce consistent long-term durable responses. The cytotoxic T-lymphocyte antigen 4 inhibitor ipilimumab has been used in metastatic melanoma and has shown an increase in survival when compared with vaccine trials; however, it has been associated with significant autoimmune toxicity (Amin & White, 2013). For melanoma patients with a BRAF V600E mutation, vemurafenib has shown a complete response rate of 6% in a phase II trial, with a median response rate of 6.7 months (Amin & White, 2013).

Compared with other treatments, high-dose IL-2 has consistently delivered durable complete responses of 10% to 15% in melanoma patients and 15% to 25% of patients with renal cell carcinoma. With properly trained practitioners and nursing staff, IL-2 should still be considered a first-line treatment for patients with renal cell carcinoma. Additionally, it is not known whether front-line treatment with another modality will change the action of IL-2 in the body (Dutcher et al., 2014). Future research might also look at the combined use of IL-2 with targeted therapies in an effort to optimize treatment (Amin & White, 2013). Further guidelines are necessary for practitioners for safe administration of IL-2 when in combination with other therapies (Dutcher et al., 2014).

## CONCLUSION

High-dose IL-2 is a chance for cure for patients with metastatic renal cell carcinoma or melanoma. The factors and profiles of patients that increase this chance for cure remain elusive. Advanced practitioners should be aware of the toxicities associated with treatment to manage patients through administration and enhance the opportunity for them to receive the maximal amount of therapy.

**Acknowledgment**

The authors thank Edward Carney, Jr., for his administrative assistance in the preparation of this manuscript.
